# Disorders of Sex Development: Management of Gender Assignment in a Preterm Infant with Intrauterine Growth Restriction

**DOI:** 10.1155/2012/587484

**Published:** 2012-03-21

**Authors:** Lucy D. Mastrandrea, Christine H. Albini, Ralph J. Wynn, Saul P. Greenfield, Luther K. Robinson, Tom Mazur

**Affiliations:** ^1^Division of Pediatric Endocrinology, Women & Children's Hospital of Buffalo, School of Medicine and Biomedical Sciences, University at Buffalo, 219 Bryant Street, Buffalo, NY 14222, USA; ^2^Division of Neonatology, Women & Children's Hospital of Buffalo, Buffalo, NY 14222, USA; ^3^Division of Pediatric Urology, Women & Children's Hospital of Buffalo, Buffalo, NY 14222, USA; ^4^Division of Genetics, Women & Children's Hospital of Buffalo, Buffalo, NY 14222, USA; ^5^Department of Psychiatry, School of Medicine and Biomedical Sciences, University at Buffalo, Buffalo, NY 14215, USA

## Abstract

We describe how a gender specialist team managed the case of a disorder of sex development in a preterm infant where definitive diagnosis and gender assignment were delayed due to complications of prematurity, anemia, and severe intrauterine growth restriction.

## 1. Introduction

Disorders of sex development (DSD) represent a class of congenital conditions in which there is atypical development of the chromosomal, gonadal, or anatomical sex. Current recommendations suggest that a multidisciplinary team be used in the management of DSDs, ensuring that families receive information in a timely, coordinated, and consistent fashion [1]. Members of the team include, at a minimum, pediatric endocrinologists, behavioral specialists, pediatric urologists, geneticists, and, in some cases, neonatologists, nurses, and social work staff [2]. Under ideal conditions, the behavioral specialist is a psychiatrist or psychologist with experience providing support to families facing the unique circumstances as well as medical and psychosocial decisions associated with DSDs. Here, we describe an unusual case of DSD complicated by prematurity and severe intrauterine growth restriction (IUGR), which limited the ability to complete comprehensive diagnostic testing. We describe how this team approach to managing gender assignment occurred in concert with the medical issues related to prematurity.

## 2. Case Report

This 0.617 kg (-3SD) infant was born at 29 2/7 weeks to a 28-year-old G1P0 mother. At 18 weeks gestation, parents were informed that the infant was female based on results of a prenatal ultrasound. Amniocentesis performed later in the pregnancy revealed a 46, XY karyotype. The parents presumed that the initial ultrasound results were incorrect and were expecting a son. In the delivery room, genital appearance was discrepant with the karyotypic sex. The phallic structure was 4 mm in length (full-term female normal 4.0 ± 1.24 mm) [3, 4]. Labia majora were fused posteriorly with no signs of hyperpigmentation or scrotalization. Labia minora were absent, and gonads were not palpable. Introital exam revealed a urethral opening at the base of the phallic structure and a dimple consistent with a vaginal opening anterior to the fusion of the labia majora. The anal opening was normally placed. Pelvic ultrasound revealed no uterus and bilateral gonadal structures consistent with testes above the inguinal ring. The infant was transferred to the neonatal intensive care unit for medical management of prematurity, respiratory distress syndrome, and anemia. No gender was assigned, and all staff was instructed to refer to the infant as “baby” or “infant.” 

Labs drawn on day 1 of life were as follows: repeat karyotype 46, XY, testosterone 239 ng/dL (normal 37–198 ng/dL, premature infant male; Kaleida Health Laboratory, Buffalo, NY) and 17-hydroxyprogesterone 122 ng/dL (normal 124–841, premature infant 26–28 weeks, day 4; Esoterix, Raritan, NJ). Additional labs to aid in the establishment of the DSD were not obtained because of the infant's low hematocrit and small blood volume. Anatomical studies including vesicoureterogram were deferred due to severe IUGR. Weekly testosterone levels were drawn to identify the expected postnatal rise in testicular testosterone at which time the plan was to obtain testosterone/dihydrotestosterone levels to address the diagnostic possibility of 5*α*-reductase deficiency. This strategy was devised by the team to maximize the likelihood of making a diagnosis at the time of testosterone surge while limiting blood draws. On day 38 of life, the infant developed presumed sepsis and expired. At the time of death, no gender had been assigned, and further testing to evaluate for 5*α*-reductase deficiency could not be performed as testosterone levels remained <20 ng/dL.

### 2.1. Clinical Case Management

On day one of life, the pediatric endocrinologist met with the parents and advised that gender assignment would be deferred while the diagnostic evaluation was pursued. The parents were informed that their child would be referred to as “baby” or “infant” until gender was assigned. In addition, they were told that a psychologist experienced in helping families and children born with these conditions would be contacted immediately and would meet with them. On day 2 of life, the psychologist met with the parents for the purpose of (1) reinforcing the recommendations of the medical team, (2) describing typical development of the sexual-reproductive system and how their baby was different, (3) agreeing to serve as a liaison between the family and the medical team, and (4) assuring them that if they decided on a gender different from the team's recommendation that this would not compromise their child's care. The parents were engaged as an integral part of the decision-making team. The parents requested that all information relevant to gender assignment be given to them in composite rather piecemeal and decided that the psychologist would be the team member to relay information to them. 

At session two on day 11 of life, the psychologist reviewed the information previously discussed and presented a detailed description of the DSD diagnoses under consideration. The parents were informed of the tests necessary to discriminate between diagnoses and the reasons that the team was unable to obtain all of the needed blood for testing. They were informed that a stepwise approach to diagnostic testing would be needed because of their child's medical condition. The parents agreed to delay gender assignment, with the team keeping open the possibility of either a male or female gender assignment. 

Prior to the third session, the psychologist organized a meeting with the medical team to discuss the parents' requests, review the general status of the infant, and discuss strategies to establish the DSD diagnosis. In addition to the psychologist, the team members at this meeting included the neonatologist, pediatric endocrinologist, and urologist. A systematic and stepwise approach towards diagnostic testing was developed taking into consideration that biochemical testing was limited by the infant's size and hemodynamic status. Session three took place on day 17 of life and included the parents, psychologist, pediatric endocrinologist, and neonatologist. The family was briefed as to the medical status and concerns that the neonatologist had related to their infant, which included persistent anemia, hypotension, and respiratory distress. The parents were interested in clarifying the DSD diagnosis; understandably, at this stage in the infant's admission, the parents were focused primarily on the medical condition of their child. The pediatric endocrinologist discussed the most likely DSD diagnoses (androgen insensitivity syndrome versus 5*α*-reductase deficiency) based on the physical exam findings and biochemical studies to date. The endocrine testing necessary to aid in the DSD diagnosis was reviewed. The parents elected to pursue biochemical testing for 5*α*-reductase deficiency while delaying genetic testing for androgen insensitivity syndrome. 

The final meeting occurred shortly after the infant's death. The parents met with the psychologist, pediatric endocrinologist, and neonatologist. The purpose was to finalize the gender assignment by giving the baby a name and to review the preliminary autopsy report. Additional genetic testing was described that might clarify the DSD diagnosis and aid in describing future risk for the family. The family elected to assign a male gender to their child, indicating that this decision was based on the initial karyotype. They consented to further postmortem genetic testing to address the DSD. While no specific queries were made, it was the impression of the multidisciplinary team that the parents were satisfied with the team's handling of this case. Mutational analysis performed to evaluate for androgen insensitivity syndrome demonstrated that there was no disease-associated mutation identified in exons 1–8 of the androgen receptor gene (GeneDx, Gaithersburg, MD). Interestingly, although the family consented to the testing, when they were contacted to review the results, they elected not to pursue further counseling.

## 3. Discussion

Disorders of sex development (DSD) are a class of congenital conditions involving anomalies of the sex chromosomes, gonads, reproductive system, and genitalia. In certain DSD diagnoses, the external genitalia are phenotypically incongruous with the genetic sex. In such cases, gender assignment management is best handled by a team of experts [2]. The DSD presented here is consistent with an undervirilized 46, XY individual. The broad differential diagnosis in this category include rare causes of congenital adrenal hyperplasia (such as StAR mutations, 17-hydroxysteroid dehydrogenase mutations, 3*β*-hydroxysteroid dehydrogenase mutations), androgen insensitivity syndrome (complete or partial), 5*α*-reductase deficiency, ovotesticular DSD, gonadal dysgenesis, and single gene mutations related to testicular development (SRY, ST1, SF1) [2]. However, it is recognized that in as many as 50% cases of 46, XY DSD, no definitive diagnosis is made, with 30% of those having a codiagnosis of prematurity/IUGR [5]. Even amongst individuals with an established clinical diagnosis, the genetic and biochemical milieu is complex. In fact, in a study of individuals diagnosed clinically with either complete (CAIS) or partial androgen insensitivity (PAIS), androgen receptor (AR) binding was normal in 7% and 64% of CAIS and PAIS patients, respectively, and AR gene sequencing was normal in 17% (CAIS) and 77% (PAIS) patients for whom genetic mutational analysis was performed [6]. In addition, AR binding was normal in 3 of 69 individuals for whom exon screening of the AR gene revealed a mutation [6]. Based on the clinical presentation of our case, there was a high likelihood that a definitive diagnosis would not be established. However, in order to provide the family with the most complete information regarding long-term outcomes with respect to gender assignment, as well as risk to future children, the team pursued the biochemical and genetic testing necessary to establish a diagnosis for the family.

In the presented case, the karyotype, elevated testosterone level at birth, and presence of intra-abdominal testes are consistent with male sex; however, the limited virilization of the external genitalia virilization brings this assignment into question. In fact, if the prenatal karyotype had not been performed, this infant may not have been recognized as having ambiguous genitalia and may have been assigned female gender. Gender assignment is dependent on a number of factors including the diagnosis, the appearance of the genitalia, options for surgical intervention, potential for fertility, family outlook, as well as long-term treatment, and outcome-based evidence regarding satisfaction with gender assignment within different DSD diagnoses [2]. It was reported previously that individuals diagnosed with 46, XY CAIS established an adult female gender identity [7]. However, 3 recently reported cases of 46, XY CAIS individuals initially assigned as females self-changed gender to male [8–11]. These cases highlight the unanswered questions regarding the role of intrauterine brain androgenization and development of gender identity, particularly since effects of prenatal and postnatal androgens are presumed to be absent in those with CAIS. Individuals with PAIS are dissatisfied with their gender assignment 25% of the time, regardless of the initial assignment either male or female [12]. A majority of individuals with 5*α*-reductase deficiency assigned female gender at birth transition to the male gender after puberty, and all those assigned male gender at birth live as males [13]. The outcome evidence related to satisfaction with gender assignment for other 46, XY DSDs is limited by small patient numbers and difficulties with long-term followup. In our case, diagnosis of DSD was significantly hindered by the infant's prematurity, extreme IUGR, and hemodynamic status. A stepwise diagnostic approach was taken to minimize the risk of compromising the infant's clinical status, while, at the same time, striving to arrive at a diagnosis that would aid the family in assigning gender [14].

## 4. Conclusion

We present a case in which management was carried out by a multidisciplinary team consisting of pediatric endocrinologists, neonatologists, a pediatric urologist, a geneticist, and a psychologist specializing in the management of DSD. The psychologist served as the team leader coordinating communication between the parents and the medical professionals. The team provides the family with a comprehensive explanation of normal and abnormal sexual differentiation which becomes a template upon which they can understand the DSD diagnosis and arrive at a decision regarding gender assignment. In addition, when available, scientifically validated outcome data is shared with the family regarding specific diagnoses [2]. 

 In most cases, an infant born with a DSD is considered a medical “emergency”; every attempt is made to arrive at a medical diagnosis in a timely fashion. A lack of diagnosis is perceived as anxiety provoking for families and caregivers when faced with an infant for whom gender assignment is unclear. Based on our experience, we would argue that when information related to patient care is relayed in a consistent fashion, this anxiety may be alleviated. In our case, the decision to assign gender was deferred for over 5 weeks, with the final decision (male) only being made by the parents upon the infant's untimely death. 

This case teaches us an important lesson about the management of disorders of sex development by demonstrating that, with support and education, gender assignments can be delayed while diagnostic workup is being pursued. We believe that this team-based strategy can be employed in other complicated medical cases in which a diagnosis or treatment regimen must be deferred. Given that the most recent consensus statement encourages the team approach as the standard of care for management of disorders of sex development [2], randomized studies to address whether parental anxiety is minimized by this method are unlikely to be feasible. Validated questionnaires to address the concerns of families related to the process of education, counseling, diagnostic testing, and decision making will be an important component to test our success in this field. Data gleaned from clinical trials assessing parental stress and developmental stage at which children are diagnosed with disorder of sex development may guide the development of such questionnaires in the future [15].

## Abbreviations

DSD: Disorders of sex development
IUGR: Intrauterine growth restriction.
